# Long-Term Outcomes of Transcatheter Aortic Valve Replacement of Degenerated Aortic Valve Homograft

**DOI:** 10.1016/j.jscai.2025.102631

**Published:** 2025-03-25

**Authors:** Shubhadarshini Pawar, Vivek Patel, Jair Basantes, Ofir Koren, Tarun Chakravarty, Mamoo Nakamura, Wen Cheng, Sabah Skaf, Hasan Jilaihawi, Raj R. Makkar, Aakriti Gupta

**Affiliations:** Department of Interventional Cardiology, Smidt Heart Institute, Cedars-Sinai Medical Center, Los Angeles, California

**Keywords:** aortic valve homograft, long-term outcomes, valve-in-valve transcatheter aortic valve replacement

Replacement of an aortic root homograft is a challenging surgical procedure associated with low success rates, significant postoperative complications, and high mortality rates.^1^ Alternative to surgical treatment, valve-in-valve transcatheter aortic valve replacement (ViV-TAVR) for degenerated aortic valve homograft is complex due to the stentless nature of the bioprosthesis, which presents challenges in anchoring.^1^^,^^2^ Studies have shown that stentless ViV-TAVR has twice the likelihood of malpositioning compared to stented ViV-TAVR. Factors contributing to this include the absence of fluoroscopic markers from a stent frame or sewing ring, noncalcified leaflets, and prominent calcification within the aortic root. Additional concerns include the proximity of the coronary ostia, which can complicate valve deployment and increase the risk of coronary obstruction.^3^^,^^4^ Oversizing has also been identified as a significant issue, especially in failing homograft, as there are no established guidelines for device sizing in these stentless bioprostheses. Coronary obstruction is also a more frequent complication in stentless ViV-TAVR compared to stented ViV-TAVR. This is primarily due to the lack of a rigid stent frame and the displacement of noncalcified leaflets, which can obstruct coronary ostia.^3^^,^^4^ In contrast, stented ViV-TAVR benefits from a more stable anchoring mechanism, leading to higher procedural success rates and reduced complications such as a paravalvular leak (PVL).^2^, ^3^, ^4^ Understanding the differences between stented and a stentless homograft ViV-TAVR is essential for optimizing patient selection and procedural planning, particularly in high-risk populations. We present a long-term follow-up of a continuous series of patients at a single medical center who underwent ViV-TAVR for degenerated aortic valve homograft.

We retrospectively analyzed consecutive patients who received ViV-TAVR for failing aortic valve homograft at Cedars-Sinai Medical Center in Los Angeles between June 2013 and June 2023. Preoperative clinical data, operative details, and follow-up information were obtained from electronic medical records. A core laboratory analyzed preprocedural computed tomography images and follow-up transthoracic echocardiograms. Valve selection was based on consensus by the heart team, considering patient anatomy, homograft condition, and clinical profile. The JenaValve Trilogy valve was used in cases with aortic regurgitation (AR), and self-expandable valves were employed for patients with an annulus too large for balloon-expandable valves. The institutional review board committee approved the study protocol, and informed consent was not needed due to the retrospective nature of the study.

**Figure 1:**
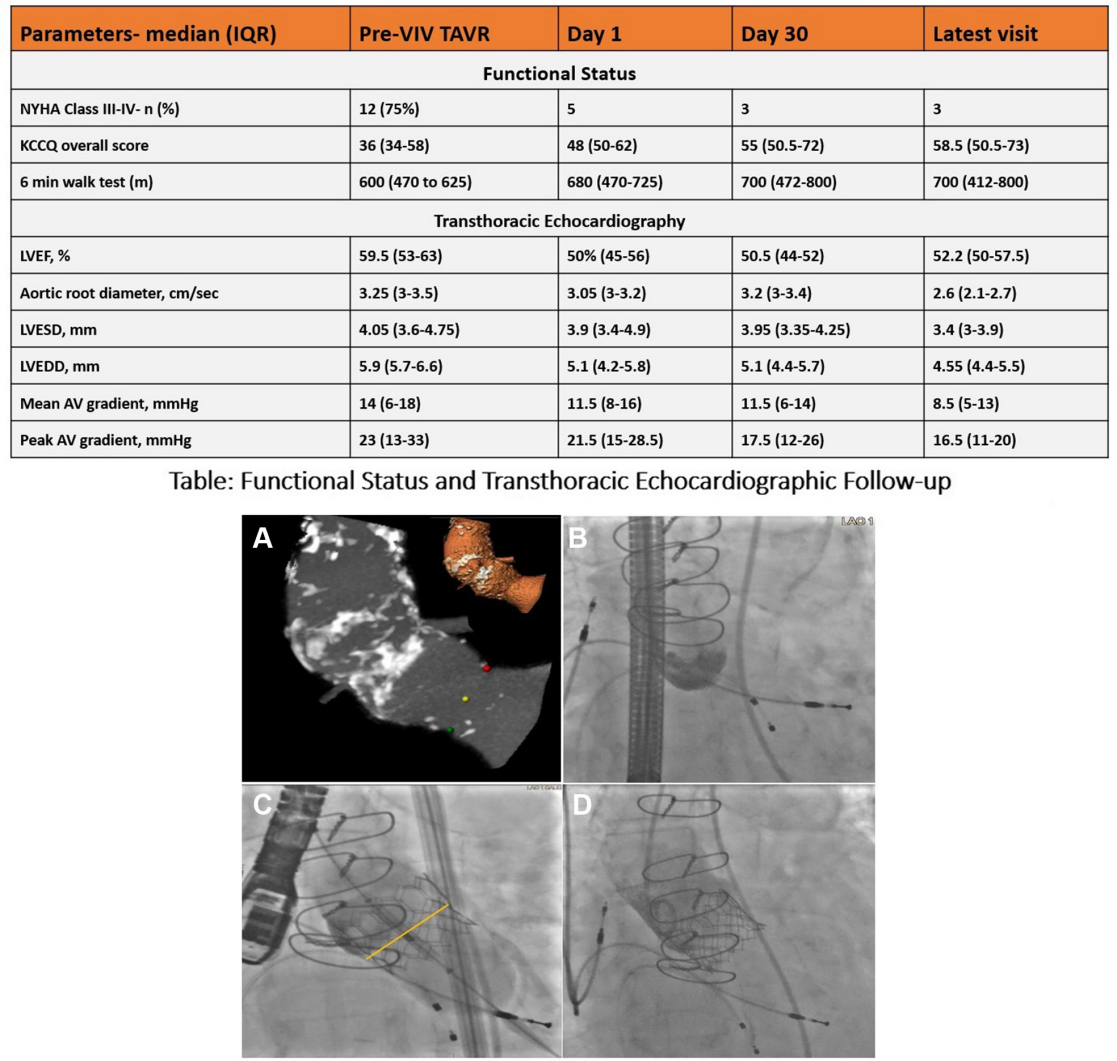
**Failed homograft transcatheter aortic valve (AV) replacement.** (**A**) Multiplanar and 3D reconstruction of the aortic root from a computed tomography scan. (**B**) No calcific landmarks. (**C**) Lower deployment of the SAPIEN 3 Ultra (yellow marker indicates annulus plane). (**D**) Fully deployed SAPIEN 3 Ultra. The table shows the functional status and transthoracic echocardiographic data. All values are median (Q1-Q3) unless specified. LVEDD, left ventricular end-diastolic diameter; LVEF, left ventricular ejection fraction; LVESD, left ventricular end-systolic diameter.

Sixteen patients with degenerated aortic valve homograft (median age 62 years; IQR, 54-74 years, 11 male) underwent ViV-TAVR (Figure 1). Of these, 5 patients (31.25%) received old-generation valves (used until the year 2016), whereas 11 patients (68.75%) received new-generation valves (utilized from 2017 to 2023). The median time from homograft aortic valve replacement to ViV-TAVR was 10.5 years (IQR, 6-14.5 years). Four patients (25%) had bicuspid aortic valve at baseline. All presented with severe symptomatic transvalvular AR, and 2 had concomitant aortic stenosis. Of the total cohort, 12 patients (75%) were categorized into New York Heart Association class III or IV, with a median Kansas City Cardiomyopathy Questionnaire score of 36 (IQR, 34-58) and a median 6-minute walk test of 600 meters (IQR, 470 to 625 meters) (Figure 1). The enrolled patients had multiple comorbidities, including atrial fibrillation in 8 (50%), coronary artery disease in 5 (31.25%), cerebrovascular disease in 6 (37.50%), and hypertension in 9 (56.25%) with median Society of Thoracic Surgeons Predicted Risk of Mortality score of 3.1 (IQR, 2.5-4.5).

Preprocedural echocardiogram reported moderate-to-severe mitral regurgitation in 8 (50%) patients and moderate-to-severe tricuspid regurgitation in 4 (25%) patients and elevated pulmonary systolic arterial pressure: 38 (24-49 mm Hg). In the preprocedural computed tomography scan, the median aortic annulus perimeter was 75.5 mm (IQR, 70-84 mm) and the aortic annulus area was 420 mm^2^ (IQR, 412-520 mm^2^). The aortic valve area was 1.79 cm^2^ (IQR, 1.29-2.45) and the sinotubular junction diameter was 27.75 mm (IQR, 25.72-30.05). The median left coronary artery height was 14.3 mm (IQR, 9.75-22.45), the right coronary artery height was 17 mm (IQR. 14.4-19.5), and 2 patients had calcified leaflets: 1 with a mild calcium burden and 1 with a moderate calcium burden. The access route was transfemoral in all patients. None of the patients required coronary protection given adequate coronary heights and measurements of sinuses and sinotubular junction. Balloon-expandable valves were used in 10 patients, whereas self-expanding valves were used in 6 patients, including 2 Evolut (Medtronic), 1 JenaValve Trilogy valve, and 3 CoreValve (Medtronic) devices. SAPIEN (Edwards Lifesciences) valve sizes ranged from 23 mm to 29 mm, with a median of 26 mm (IQR, 26-29 mm). Evolut valve sizes were 34 mm and 26 mm, JenaValve Trilogy valve was 27 mm, and CoreValve devices were 29 mm, 31 mm, and 31 mm. The median transcatheter aortic valve annulus area oversize for balloon-expanding valves was 13.85% (IQR, 10.3%-19.3%), for Evolut valves it was 17.6% and 13.5%, for the JenaValve Trilogy valve it was –3.6%, and for 3 CoreValve devices it was 15.2%, 15.7%, and 16.9%. The SENTINEL device (Boston Scientific) was utilized in 1 patient (0.06%) due to heavy calcium burden on valve leaflets.

There were no in-hospital deaths, myocardial infarctions, cardiac tamponade, or significant bleeding reported. The median follow-up duration was 3.53 years (IQR, 1.31-8.02 years). Two patients (12.50%) who received new-generation valves required pacemaker implantation for complete heart block. At the median follow-up, one patient died from a noncardiac cause. Among the old-generation valves, 1 patient (6.25%) experienced a myocardial infarction, while another patient (6.25%) had a cerebrovascular event. At the 9-year follow-up, 1 patient (6.25%) was diagnosed with prosthetic valve endocarditis. One of the patients was a 59-year-old male with a history of ischemic cardiomyopathy with left ventricular ejection fractions of 35% and a history of triple vessel coronary bypass surgery and aortic aneurysm, status post-Bentall procedure in 2013 who presented with severe AR in 2014. His annulus measured 529 mm^2^, and area-derived perimeter was 84 mm. He was treated with a self-expanding valve (31 mm CoreValve) with 20% oversizing yielding an optimal procedural result with only trace PVL. However, 2 months later, the patient presented with moderate-to-severe PVL and bioprosthetic AR and was found to have ventricular migration of the valve, even though the valve was still anchored at the annulus. As such, a ViV-TAVR with a balloon-expandable valve was performed (SAPIEN 29 mm) with optimal anchoring and no residual AR. Of the total cohort of 16 patients, the median transaortic gradient at follow-up was 8.5 mm Hg, with only 1 patient (6.25%) having a mean gradient greater than 20 mm Hg. Additionally, only 2 patients (12.5%) were noted to have a mild PVL. Overall, improved hemodynamics was seen at each follow-up visit (Figure 1).

These outcomes are consistent with previously published studies and case series.^1^^,^^3^^,^^5^ The Royal Brompton Hospital in London, UK, reported 22 high-risk patients with degenerated stentless bioprostheses or aortic homografts who underwent transcatheter aortic valve replacement (TAVR) with CoreValve devices. Two patients experienced prosthesis migration, with 1 undergoing emergency surgical AVR due to embolization. However, no in-hospital or 30-day deaths were reported. Additionally, 13.6% of patients required a new permanent pacemaker.^3^ A prior study by Sedeek et al^1^ reported 11 patients with failing homografts who underwent TAVR, with predominantly used SAPIEN 3 valves. They reported satisfactory outcomes related to the TAVR procedure, intermediate-term results, and improved hemodynamic performance. Previous research on percutaneous treatment of degenerated aortic homografts has been focused on self-expanding valves due to their ability to be removed and repositioned, considering the concern about valve embolization.^1^^,^^3^^,^^5^ The balloon-expandable SAPIEN 3 valve ensures immediate full expansion upon deployment leading to precise placement, predictable results, and effective hemodynamics. Additionally, the outer skirt design enhances sealing, helping to minimize PVL, a common concern in valve-in-valve procedures. Although our experience is limited to only 16 patients, our low mortality rate, prosthesis durability, improved hemodynamics, and acceptable TAVR procedure-related long-term clinical outcomes demonstrate that ViV-TAVR is safe in patients with failing homograft who are either at high risk of surgery or do not wish to undergo redo sternotomy.

Our study has certain limitations. First, it was retrospectively conducted at a single medical center. Second, our follow-up period and small sample size did not address ViV-TAVR prostheses durability and long-term procedure-related adverse events and outcomes.

This case series from a tertiary care valve center demonstrates that ViV-TAVR is safe, effective, and feasible in patients with degenerate aortic valve homograft and can prevent redo sternotomy. Long-term valve hemodynamics appear favorable in this single-center series.
